# Phenytoin-Associated Stevens-Johnson Syndrome and Toxic Epidermal Necrolysis Overlap in Focus: A Case Report

**DOI:** 10.7759/cureus.46075

**Published:** 2023-09-27

**Authors:** Piyush Puri, Zaid M Aslam, FNU Komal, FNU Prachi, Princy Sardana, Akshit Chitkara

**Affiliations:** 1 Internal Medicine, Adesh Institute of Medical Sciences and Research, Bathinda, IND; 2 Surgery, Ziauddin University, Karachi, PAK; 3 Internal Medicine, HCA Houston Healthcare Northwest, Houston, USA; 4 Internal Medicine, Guru Teg Bahadur Hospital, Delhi, IND; 5 Internal Medicine, Saraswathi Institute of Medical Sciences, Hapur, IND; 6 Internal Medicine, University of California Riverside School of Medicine, Los Angeles, USA

**Keywords:** side effects of medical treatment, head injury and epilepsy, toxic epidermal necrolysis (ten), stevens-johnson syndrome (sjs), phenytoin

## Abstract

Stevens-Johnson syndrome and toxic epidermal necrolysis overlap is a rare but severe cutaneous hypersensitivity reaction that can lead to death if not treated aggressively and adequately. Drug-induced hypersensitivity reactions are often related to drug exposure, with sulfonamides, anti-epileptics, fluoroquinolones, cephalosporins, and nonsteroidal anti-inflammatory drugs being the most common culprits. This case report describes a 10-year-old boy who was administered phenytoin at a local clinic to manage his seizures. This treatment led to the onset of SJS-TEN overlap, ultimately resulting in his demise.

## Introduction

Stevens-Johnson syndrome and toxic epidermal necrolysis (SJS/TEN) are severe cutaneous adverse reactions that affect mucocutaneous surfaces [1]. SJS is characterized by the detachment of less than 10% of the body surface area, whereas TEN involves the detachment of more than 30% of the body surface area. Detachments involving 10-30% of the body surface area fall within the overlap of SJS/TEN. The mortality rate for SJS-TEN ranges from 10% to 34% [2]. In the United States, the average estimated incidences of SJS, SJS/TEN overlap, and TEN among adults were 9.2, 1.6, and 1.9 cases per million individuals per year, respectively [2]. The mean adjusted mortality rates were 4.8% for SJS, 19.4% for SJS/TEN, and 14.8% for TEN [2]. Some of the identified causes of SJS/TEN include medications, mycoplasma pneumonia, herpes, hepatitis A, and vaccination [3]. The primary cause of this life-threatening disorder is drugs, with common examples being sulfonamides and anti-epileptic drugs such as phenytoin, carbamazepine, lamotrigine, phenobarbital, and allopurinol, as well as non-steroidal anti-inflammatory drugs like piroxicam, diclofenac, and nevirapine [4].

## Case presentation

**Figure 1:**
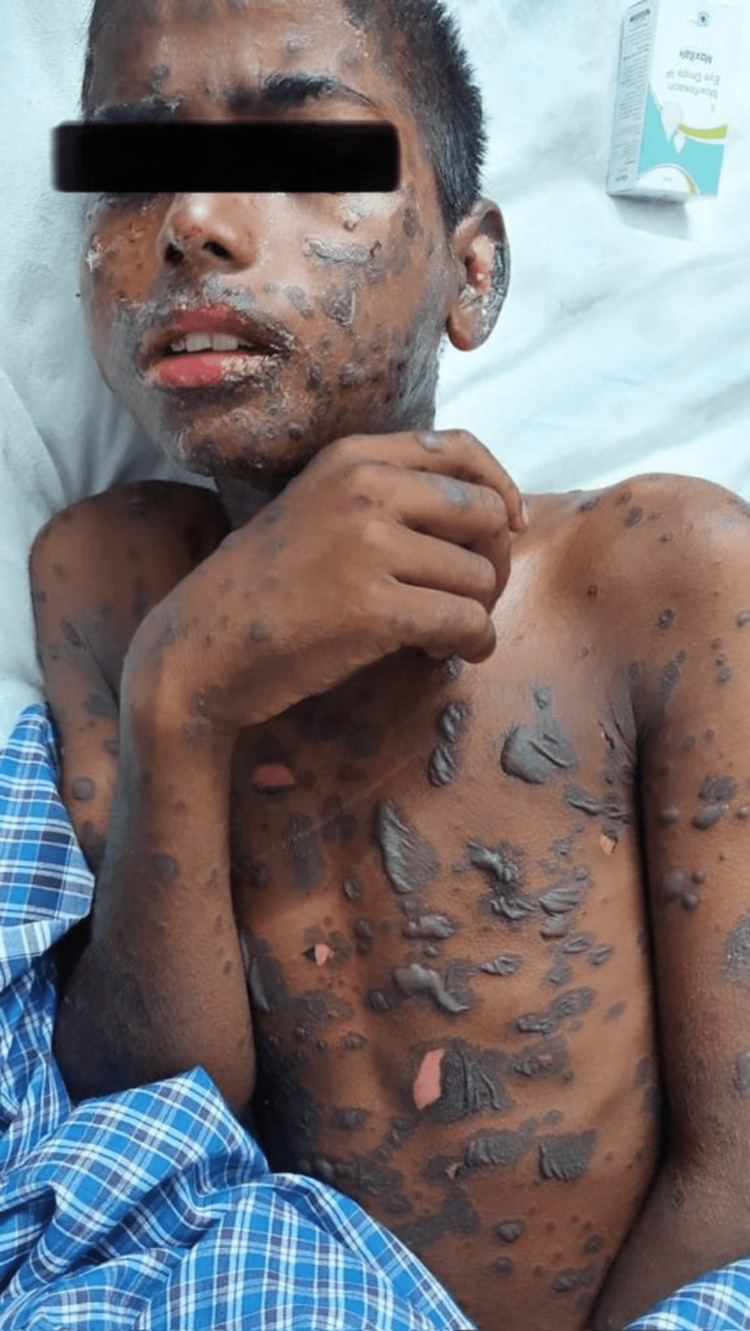
Picture depicting the involvement of body surface area with erosions and ulcers

A 10-year-old boy presented with initial symptoms of generalized itchiness, loss of appetite, fever, and fatigue, followed by multiple skin erosions, mouth ulcers, and genital ulcers. The patient had a history of a fall from a height and experienced seizure episodes 6-7 days prior. He was treated with phenytoin for seizures at a local clinic. Upon admission, the medical team recorded the patient's vital signs: blood pressure (BP) 90/43 mmHg; pulse rate 122/min; oxygen saturation (SpO2) 96%; and respiratory rate 23/min. The patient exhibited severe dehydration and received a clinical diagnosis of SJS-TEN overlap, involving 10-30% of his body surface area (BSA) (Figure 1). The patient was admitted to the ICU, where phenytoin was discontinued immediately, and a strict aseptic environment was maintained. He received supportive therapy with intravenous (IV) fluids, and his denuded skin was treated with paraffin gauze and high-dose IV dexamethasone at 100mg/day. Antibiotics were withheld due to concerns about exacerbating the condition. On Day 2, the patient's vitals remained stable, and some improvement in skin lesions was noted after discontinuing phenytoin, although not substantially. On Day 3, the patient reported ocular discomfort, prompting an ophthalmology consultation. Artificial tears were prescribed, providing mild relief. Shortly thereafter, the patient experienced breathing difficulties and desaturation, necessitating nebulization and bronchodilators. However, his condition rapidly deteriorated, requiring intubation. Laboratory findings revealed an elevated white blood cell count (Table 5) and confirmed acute kidney injury (Table 3). Treatment was initiated with cyclosporine, acetaminophen, povidone-iodine mouthwash, piperacillin-tazobactam, and intravenous fluids. A nasogastric tube was inserted for feeding support.

Naranjo criteria were used for the adverse drug reaction probability scale, where a score of 6 was achieved (Table 1).

**Table 1:** Adverse Drug Reaction Probability Scale Scoring: ≥9 = definite ADR, 5-8 = probable ADR, 1-4 = possible ADR, 0 = doubtful ADR

Question	Yes	No	Do Not Know	Score
1. Are there previous conclusive reports on this reaction?	+1	0	0	1
2. Did the adverse event appear after the suspected drug was administered?	+2	-1	0	2
3. Did the adverse event improve when the drug was discontinued or a specific antagonist was administered?	+1	0	0	1
4. Did the adverse event reappear when the drug was readministered?	+2	-1	0	0
5. Are there alternative causes that could on their own have caused the reaction?	-1	+2	0	2
6. Did the reaction reappear when a placebo was given?	-1	+1	0	0
7. Was the drug detected in blood or other fluids in concentrations known to be toxic?	+1	0	0	0
8. Was the reaction more severe when the dose was increased or less severe when the dose was decreased?	+1	0	0	0
9. Did the patient have a similar reaction to the same or similar drugs in any previous exposure?	+1	0	0	0
10. Was the adverse event confirmed by any objective evidence?	+1	0	0	0
	Total Score: 6			

The medical team calculated the Severity-of-Illness Score for Toxic Epidermal Necrolysis (SCORTEN) illness to predict mortality (Table 2). The patient's heart rate was >120/min, body surface area was >10%, and blood urea nitrogen was also >10 mmol/L giving this patient a score of 3.

**Table 2:** Severity-of-Illness Score for Toxic Epidermal Necrolysis (SCORTEN)

Risk factor (1 point each)		SCORTEN (points)	Mortality(%)
Age	≥40 years old	0-1	3.2
Heart rate	≥120 bpm	2	12.1
Cancer or hematologic malignancy	Any	3	35.3
Body surface area (BSA)	>10%	4	58.3
Blood urea nitrogen (BUN)	>10 mmol/L	≥5	90
Bicarbonate	<20 mmol/L		
Glucose	>224 mmol/L (>252 mg/dL)		

The patient was severely ill, and the condition had deteriorated. Kidney function tests showed features of acute kidney injury on Day 3 of admission (Table 3).

**Table 3:** Kidney Function Tests

Test name	Result	Normal reference range
Blood urea	52 mg/dL	15-36
Blood urea nitrogen	31.2 mg/dL	7-17
Creatinine	3.1 mg/dL	0.5-1.04
Uric acid	9.4 mg/dL	2.5-6.2
Calcium	9.3 mg/dL	8.4-10.2
Phosphorus	4.9 mg/dL	2.5-4.5
Sodium	147 mmol/L	137-145
Potassium	3.8 mmol/L	3.5-5.1
Chloride	107 mmol/L	98-107

LFT was also deranged (Table 4).

**Table 4:** Liver Function Tests A/G ratio: albumin/Globulin ratio; AST: aspartate aminotransferase; ALT: alanine aminotransferase

Test name	Result	Normal reference range
Total protein	9.1 g/dL	6.5-7.8
Albumin	4.3 g/dL	3.9-5.0
Globulin	4.8 g/dL	2.0-3.5
A/G ratio	0.9	1.5-2.5
Gamma-glutamyl transferase	80 U/L	12-38
AST	322 U/L	8-48 U/L
ALT	288 U/L	7-55 U/L

A complete blood count indicated leukocytosis (Table 5).

**Table 5:** Complete Blood Count MCH: mean corpuscular hemoglobin; MCV: mean corpuscular volume; MCHC: mean corpuscular hemoglobin concentration; RDW-CV: red cell distribution width; ESR: erythrocyte sedimentation rate.

Test name	Result	Normal Reference Range
Hemoglobin	11 g/dL	12.0-15.0
Hematocrit	50.0%	36-46
MCV	98 fL	83-101
MCH	35.8 pg	27-32
MCHC	39.3 g/dL	31.5-34.5
RDW-CV	11.8%	11.5-14.5
ESR	54 mm/hr	<20
C-reactive protein	9.2 mg/dL	<5.0
Total WBC count	16,700/mm^3^	4,000-11,000
Neutrophils	82%	40-70
Lymphocytes	15%	20-36
Eosinophils	2%	01-06
Monocytes	1%	02-08
Basophils	0%	00-02
Total platelets count	68,000/mm^3^	1.5-4.5
Absolute lymphocyte count	6.420/uL	0.600-4.100
Absolute neutrophil count	9.320/uL	2.000-7.800

Due to low blood pressure (70/46 mmHg), the patient was placed on inotropic support on the 4th day using norepinephrine. Unfortunately, on the 5th day of admission, the patient succumbed to the side effects of the drug.

## Discussion

The underlying pathophysiology of SJS-TEN involves keratinocyte apoptosis, leading to epidermolysis and subsequent blistering [5]. Cytotoxic T-cell lymphocytes, found in the blister fluid of TEN patients, are believed to trigger a cascade of intracellular enzyme activation that results in apoptosis-a series of programmed reactions leading to cellular changes and ultimately cell death [6]. Among antiepileptics, phenytoin and carbamazepine are reported as the most common culprits [7]. The Food and Drug Administration (FDA) is currently investigating the potential link between the HLA-B 1502 allele and phenytoin-induced Stevens-Johnson syndrome (SJS) in Asian patients [5]. This suggests a potential genetic predisposition to SJS in specific populations, emphasizing the importance of further research to understand the role of genetic factors in susceptibility to this condition [8].

Stevens-Johnson syndrome is primarily diagnosed through clinical evaluation [9]. A skin biopsy can confirm the diagnosis and rule out other possible causes of the reaction. Additional tests, such as skin culture, oral culture, and blood tests, can help rule out other causes, including infectious etiologies [10]. Several serum markers have been explored for early detection of TEN and monitoring disease progression [11]. These include soluble CD40 ligand, Fas ligand, granulysin, granzyme B, serum high mobility group protein B1, alpha-defensins 1-3 in blister fluid, Bcl-2 expression in dermal infiltrates, thymus and activation-regulated chemokine, and glutathione-S-transferase-pi expression. IL-15 has been found particularly useful for predicting disease severity and monitoring prognosis [12,13]. Histological examination of cryosections or formalin-fixed sections of the skin can confirm extensive necrosis involving all layers [14]. To rule out autoimmune blistering disorders, direct immunofluorescence staining should be performed, as it aids in detecting the presence or absence of immunoglobulin and complement deposition in the epidermis or at the epidermal-dermal junction.

The management of SJS-TEN overlap necessitates prompt hospitalization in an intensive care unit or a burn unit [15]. Nonetheless, it is worth noting that silver sulfadiazine, a sulfonamide, has been associated with toxic epidermal necrolysis. The primary treatment measure for Stevens-Johnson syndrome is discontinuing the use of the triggering medication. Supportive care, including fluid replacement and nutrition, is important. Widespread skin loss in SJS-TEN leads to extensive dehydration; therefore, fluid replacement is of utmost importance [16]. Cool and wet compresses may be used to soothe blisters. Certain medications can be administered to provide symptomatic relief; for example, pain medication can be used to reduce discomfort, topical steroids can help reduce inflammation of the eyes and mucous membranes, and systemic antibiotics can be used only if there are signs of infection or sepsis but not prophylactically. Etanercept and cyclosporine have been shown to be effective, as suggested by some studies [17,18].

## Conclusions

SJS-TEN overlap is a serious condition that should be diagnosed early in the course of treatment. Therefore, clinicians should be proactive and aware of the clinical symptoms of this condition. Early diagnosis, careful monitoring of complications, and supportive care play major roles in the treatment of SJS-TEN overlap. Care should be taken with offending drugs, such as carbamazepine, phenytoin, ibuprofen, piroxicam, and amoxicillin.
